# Effects of esters’ cetylated fatty acids taping for chronic neck pain with mobility deficit in patients with breast cancer

**DOI:** 10.1007/s00520-022-07497-2

**Published:** 2022-12-14

**Authors:** Rosanna Izzo, Mariasole Rossato, Germano Tarantino, Nicola Mascolo, Mauro Puleio

**Affiliations:** 1 Complex Operating Unit “Rehabilitation Activities,” Torre del Greco Hospital, ASL Napoli 3 Sud, Naples, Italy; 2 Scientific Department Pharmanutra Spa, Pisa, Italy; 3 Complex Operating Unit “Oncology,” Torre del Greco Hospital, ASL Napoli 3 Sud, Naples, Italy

**Keywords:** Breast cancer, Neck pain, Rehabilitation, Cetylated fatty acids

## Abstract

**Purpose:**

To evaluate the effects of a protocol treatment based on inelastic adhesive tape with cetylated fatty acids (CFAs) esters in breast cancer survivors with chronic neck pain.

**Methods:**

In this observational study, patients have been visited for chronic neck pain using numeric rating scale (NRS) for pain assessment, Neck Disability Index (NDI) for disability caused by neck pain, and range of movement (ROM) measures for cervical mobility. Scales have been performed at T0, after 15 days of treatment (T1) and successively after 15 days of stop treatment (T2). Patients have been treated with an inelastic adhesive tape with CFA esters (Cetilar® Tape, Pharmanutra Spa, Italy) positioned, 8 h/day for 15 days, on specific anatomic sites (upper trapezius, paravertebral cervical muscles, sub-occipitals, and/or levator scapulae muscles).

**Results:**

Forty-five patients were included in the study. A statistically significant reduction in pain has been reported from T0 to T1 and maintained at T2 (*p* < 0.05); a statistically significant improvement in the mobility of the cervical spine, as evidenced by ROMs, and in disability, as resulted by Neck Disability Index, have been reported from T0 to T1 and maintained at T2; moreover, ROM at T0 correlates inversely and statistically significantly with NRS and all NDI variables at T0, similarly at T1 and T2 (*p* < 0.001).

**Conclusions:**

CFA ester taping is a simple, effective, and side-effect-free treatment in order to reduce pain and improve cervical mobility in breast cancer survivors with chronic neck pain.

## Introduction

Breast cancer is the most prevalent and incident cancer in women, with 2.3 million of new cases reported in 2020 worldwide, and 685,000 deaths during the same year [1, 2]. As reported by WHO (*World Health Organization*), during 2020, about 7.8 million women have survived breast cancer diagnosed in the last 5 years, thus representing the form of cancer with the highest prevalence worldwide [1]. Surgery, chemotherapy, radiotherapy, and hormonal therapy for breast cancer lead to many consequences, such as inflammation and tissue adherence, causing pain and altering the patients’ quality of life (QoL) [3, 4]. The more frequent rehabilitation problems in women with breast cancer are as follows [5–9]:

- Reduction in upper limb strength and mobility
- Upper limb lymphedema
- Chronic pain (benign, regarding joints, and muscles)
- Fatigue
- Osteopenia and osteoporosis

Chronic musculoskeletal pain (CMP) is reported in 60% of breast cancer survivors: shoulder, neck, arm, and thorax are the most common sites of chronic pain, negatively affecting the quality of life [10–12]. The origin of musculoskeletal pain in these patients is certainly multifactorial, as a consequence of postural post-surgery disorders, chemo and radiotherapy treatments that can worsen adhesions and consequently postural disorders, but also hormonal therapies that are associated with diffuse musculoskeletal pains (aromatase inhibitor–associated musculoskeletal syndrome (AIMSS)), and finally the psychological impact which constitutes a predisposing factor for the chronicity of pain [3, 4, 10–12]. A multidisciplinary approach is proposed, including stretching, aerobic exercises, strength exercises, acupuncture, and manual therapy, not only to reduce pain and prevent chronicity, but also to improve QoL [11, 13, 14]. About CMP, neck pain with myofascial trigger points in upper trapezius muscle is widely reported in literature in breast cancer survivors [15–18].

As reported by Blanpied et al. [19], neck pain can be classified into the following categories: (1) neck pain with mobility deficits, (2) neck pain with movement coordination impairments, (3) neck pain with headaches (cervicogenic headache), and (4) neck pain with radiating pain. For chronic neck pain with mobility deficits, guidelines [20–22] provide a multimodal approach including thoracic and cervical manipulation, mixed exercise for cervical/scapulothoracic regions (including stretching exercises, strengthening, postural training), dry needling, laser, and intermittent mechanical/manual traction. A pharmacological approach could be proposed in case of exacerbation of pain, including topical NSAIDs, paracetamol and/or NSAIDs in initial phases, opioids, and muscle-relaxant drugs for a short period [20–22].

Cetylated fatty acid (CFA) esters are acids of vegetable origin esterified with cetyl alcohol that, after topical administration, are rapidly absorbed by a passive permeation, favored by the lipid nature of the cell membranes. CFA mechanism of action includes synovial membrane protection and cell membrane stabilization, promoting normal flexibility and mobility, obtaining a reduction in pain and an increase in joint fluidity and lubrication [23–29]. CFAs in topical formulations are useful in improving joint mobility, functionality, and strength, as well as reducing pain symptoms in absence of side effects in different clinical settings [30–35].

The aim of our study is to evaluate the effects of a protocol treatment in chronic neck pain with mobility deficits using inelastic adhesive tape with CFA esters in breast cancer survivors.

## Materials and methods

This observational quasi-experimental study has been performed at the Oncological Rehabilitation Clinic, Torre del Greco Hospital (Naples, Italy), between June and December 2021. The study has been performed in accordance with the *Strengthening The Reporting Of Observational Studies In Epidemiology (Strobe) Guidelines*.

### Population—process

The study recruited adult women with breast cancer and suffering from neck pain who came to our clinical rehabilitation unit and were evaluated by a team of physiatrists. Women with the following characteristic: neck pain for more than 90 days, breast cancer stadium I–IIIa, surgically treated with quadrantectomy or mastectomy associated to axillary lymph node dissection and/or sentinel lymph node biopsy, and patients who have finished chemo and/or radiotherapies were included. Patients with recent neck trauma, neck surgery, NSAIDs or muscle-relaxants drugs in the last 3 weeks, active radicular pain, and active arthritis with involvement of the cervical spine were excluded from the study.

### Assessment

Patients with breast cancer underwent a physical examination for chronic neck pain assessment, based on the following scales and measures:

- Numeric rating scale (NRS) for pain assessment [36]
- Neck disability index (NDI) for disability caused by neck pain [37, 38]
- Range of movement (ROM) measures (flexion, extension, lateral flexion, and rotation), using a goniometry, for the mobility of the cervical spine

Scales have been performed at the first visit (T0), after 15 days of treatment (T1) and successively 15 days after the interruption of the treatment (T2). NRS requires the patients to rate their pain on a scale 0–10 where 0 is no pain and 10 is the worst pain imaginable [29]. NDI is a standard instrument for measuring self-rated disability due to neck pain; it is composed by 10 items scores from 0 to 5, its maximum score is 50, and the obtained score can be multiplied by two to produce a percentage score (minimal disability 0–20%, moderate 21–40%, severe 41–60%, disabling 61–80%, bedridden 81–100%) [30, 31]. All patients practiced cervical spine X-rays before starting the treatment, in order to confirm the benign cause of chronic neck pain.

### Treatment

**Fig. 1:**
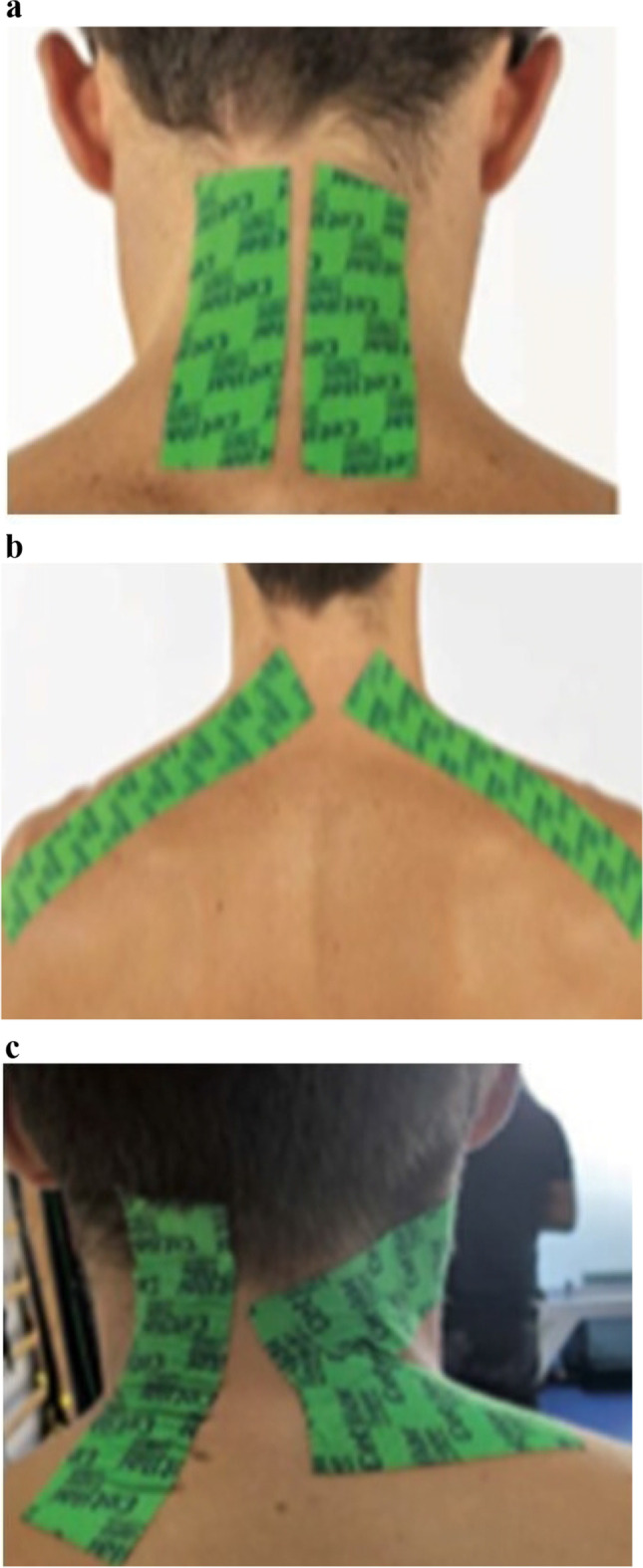
**a** Tape placed on the paravertebral muscles of the neck. **b** Tape placed on the upper trapezius muscle. **c** Tape placed on sub-occipitals and levator scapulae muscles

All patients are treated using a protocol treatment based on inelastic adhesive tape with CFA esters (Cetilar® Tape, Pharmanutra Spa, Italy) positioned, 8 h/day for 15 days, on specific anatomic sites (upper trapezius, paravertebral cervical muscles, sub-occipitals, and/or levator scapulae muscles), depending on the pain, following the model attached (see Fig. 1a–c). One tape strips of about 12–14 cm in length have been used, depending on the physical conformation of the patient; tapes are for single use and not reusable.

All patients gave their written informed consent to treatment, and all authors were instructed to protect the privacy and the study procedures according to Helsinki Declaration; the study was approved by our Local Institutional review board (ASL NA3SUD Ethics Committee).

### Statistical analysis

Analysis SPSS program has been used to analyze statistical data; Student *t* test has been used to evaluate data pre and post treatment; Pearson’s correlation has been used for data correlation; *p* value < 0.05 has been considered statistically significant. This observational quasi-experimental study has not been registered on Clinical Trials Registry, because it is not an intervention trial, and reflects normal clinical practice.

## Results

**Fig. 2:**
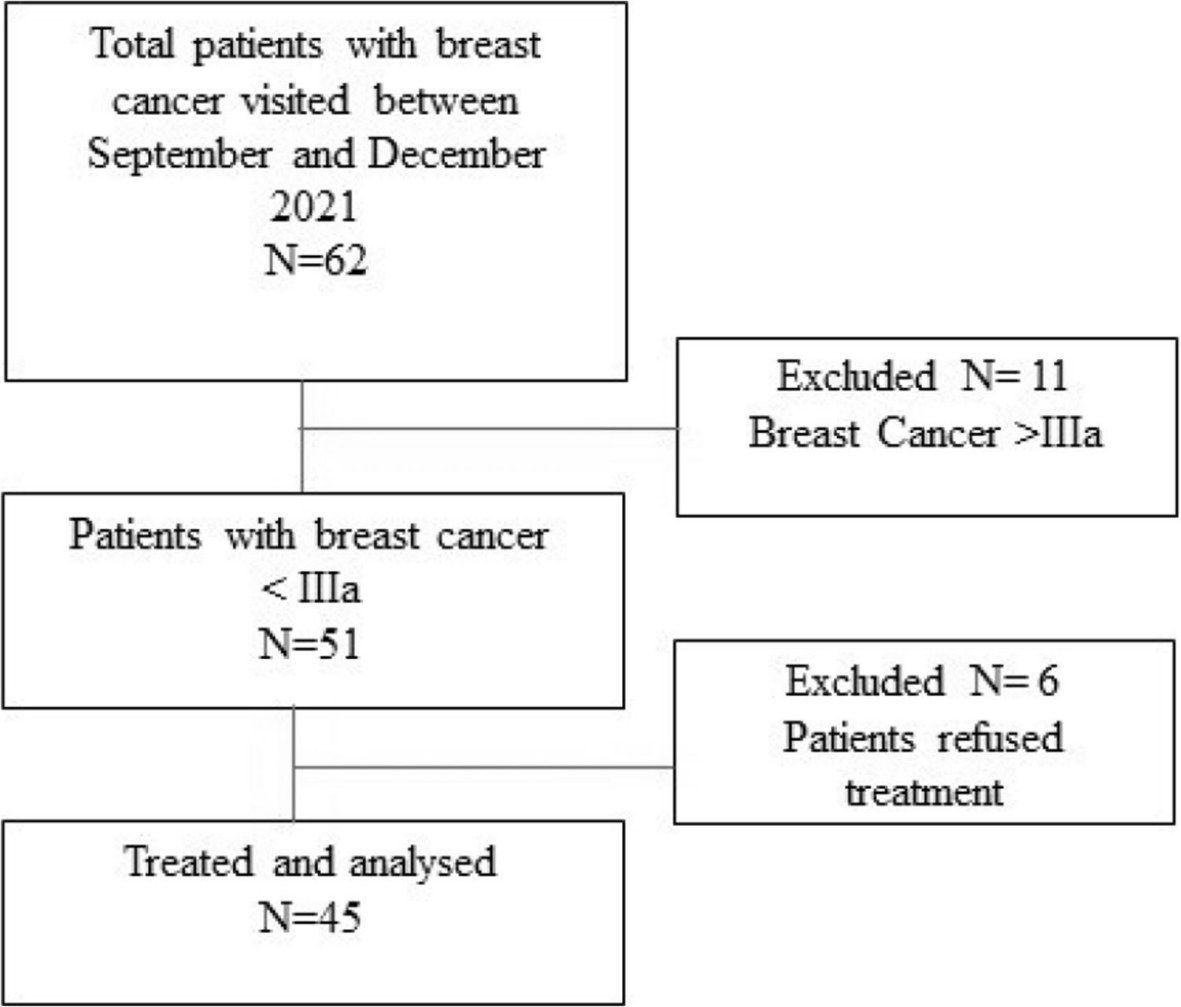
Study flow-chart: from a total of 62 patients visited between September and December 2021, 45 were recruited in the study

A group of 62 patients with breast cancer and neck pain were visited in the period between June and December 2021: 11 patients had a stage disease > IIIa, so they were excluded from the study, while among the remaining 51 patients, 6 refused the treatment; therefore, 45 patients were finally included in the study (Fig. 2). In Table 1 are summarized the sample’s demographic characteristics. No adverse reactions have been reported in patients treated. The average reported age of patients was 56.2 years (range 41–64), with an average weight of 69.5 kg (range 55–89 kg); 38 patients had undergone mastectomy plus lymphadenectomy, while 7 only received quadrantectomy; 38 patients were treated with both chemotherapy and radiotherapy (Table 1).

**Table 1:** Sample’s demographic characteristics and clinical

Number of patients	(*n* = 45)
Average Age	56.2 (41–64) years
Average weight	69.5 kg
Stadium breast cancer	I 7
	II 25
	IIIa 13
Surgery	Quadrantectomy 7
	Unilateral mastectomy 0
	Mastectomy + lymphadenectomy 38
Time since surgery	< 12 months 21
	> 12 months 24
Oncologic treatment	Chemotherapy 0
	Radiotherapy 7
	Both 38
	Hormonal therapy 39
Menopause	Pre menopause5
	Post Menopause40

**Fig. 3:**
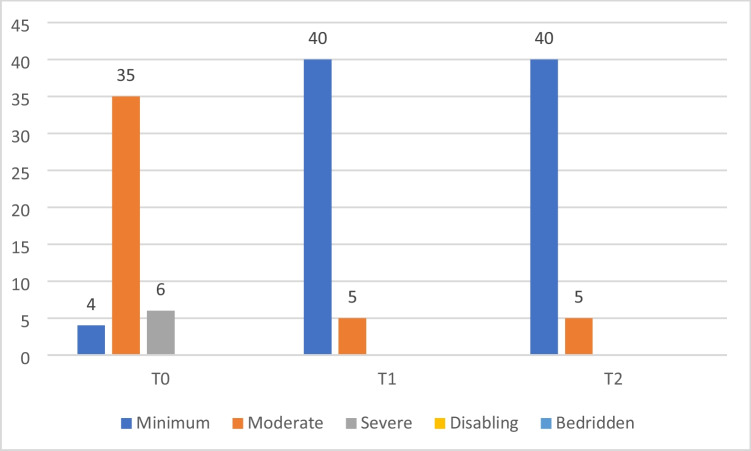
Disability neck index (NDI) at T0, T1, and T2: results distributed in minimal disability (0–20%), moderate (21–40%), severe (41–60%), disabling (61–80%), bedridden (81–100%)

Table 2 shows the average data about pain (NRS scale), disability (DNI scale), and joint mobility (ROM) at times T0, T1, and T2. A statistically significant reduction in pain on the NRS scale has been reported from T0 (6.1 ± 1.2) to T1 (2.3 ± 0.7) and maintained at T2 (2.4 ± 0.8) (*p* < 0.05). The pain reduction has been accompanied by a statistically significant improvement in the mobility of the cervical spine as evidenced by ROMs from T0 to T1 and maintained at T2: improvements have been reported in all directions, and more specifically in *flexion* (40.5 ± 3.5° at T0/53.2 ± 2.7° at T1/54.1 ± 2.2° at T2, *p* < 0.05), *extension* (42 ± 2.8° at T0/52.5 ± 1.9° at T1/53.3 ± 1.8° at T2, *p* < 0.05), *rotation* ( 40.3 ± 1.8° at T0/55.1 ± 2.4° at T1, 55.7 ± 2.3° at T2, *p* < 0.05), and *lateral flexion* (30.1 ± 1.6° at T0/45.4 ± 1.6° at T1/45.3 ± 1.8° at T2, *p* < 0.05). Moreover, a statistically significant improvement of the Neck Disability Index has been reported from T0 to T1 (*p* < 0.05), as evidenced by the reduction in moderate disability from T0 to T1 (35/45 at T0 vs 5/45 at T1) and severe disability from T0 to T1 (6/45 vs 0/45), maintained at T2, and an increase in mild disability (4/45 vs 40/45) (Fig. 3 and Table 2).

**Table 2:** Main results about pain (NRS scale), disability (DNI scale), and joint mobility (ROM) at T0, T1, and T2

	T0	T1	T2
NRS	6.1 ± 1.2	2.3 ± 0.7	2.4 ± 0.8
DNI	Minimum (0–20%) *N* = 4	Minimum (0–20%) *N* = 40	Minimum (0–20%) *N* = 40
	Moderate (21–40%) *N* = 35	Moderate (21–40%) *N* = 5	Moderate (21–40%) *N* = 5
	Severe (41–60%) *N* = 6	Severe (41–60%) *N* = 0	Severe (41–60%) *N* = 0
	Disabling (61–80%) *N* = 0	Disabling (61–80%) *N* = 0	Disabling (61–80%) *N* = 0
	Bedridden (81–100%) *N* = 0	Bedridden (81–100%) *N* = 0	Bedridden (81–100%) *N* = 0
ROM	Flexion 40.5 ± 3.5°	Flexion 53.2 ± 2.7°	Flexion 54.1 ± 2.2°
	Extension 42 ± 2.8°	Extension 52.5 ± 1.9°	Extension 53.3 ± 1.8°
	Rotation 40.3 ± 1.8°	Rotation 55.1 ± 2.4°	Rotation 55.7 ± 2.3°
	Lateral flexion 30.1 ± 1.6°	Lateral flexion 45.4 ± 1.6°	Lateral flexion 45.3 ± 1.8°

*NRS* numeric rating scale, *DNI* disability neck index, *ROM* range of movement

Table 3 shows the results obtained correlating ROM with NRS and DNI scale using Pearson test: interestingly, ROM at T0 correlates inversely and statistically significantly with NRS at T0, similarly at T1 and T2 (*p* < 0.001), indicating pain reduction improves cervical spine mobility; in addition, there is an inverse and statistically significant correlation between ROM and all NDI variables (*p* < 0.001), indicating mobility improvement is associated with disability reduction.

**Table 3:** Correlation results between ROM and DNI, ROM and NRS at T0, T1, and T2, using Pearson test

	ROM
DNI	T0 T1 T2
	*R* = − 0.664 *R* = − 0.659 *R* = − 0.662
	*p* < 0.001 *p* < 0.001 *p* < 0.001
NRS	T0 T1 T2
	*R* = − 0.677 *R* = − 0.664 *R* = − 0.664
	*p* < 0.001 *p* < 0.001 *p* < 0.001

*DNI* disability neck index, *NRS* numeric rating scale, *ROM* range of movement

## Discussion

Many breast cancer survivors will experience physical and psychological sequelae that affect their everyday lives. The side effects of treatment as well as inactivity secondary to treatment can impair activity and participation, decrease independence, and affect quality of life [7]. Chronic neck pain in breast cancer survivors is well reported in literature [10, 15–18]: Caro-Moran et al. [17] evidenced how pressure pain threshold on trapezius and dorsal muscles are lower on the side of surgically breast treated than on the contralateral side, showing greater pain in this area. Fernandez-Lao et al. [18] reported myofascial trigger points in neck and shoulder muscles and widespread pressure pain hypersensitivity in patients with postmastectomy pain: specifically, the local and referred pain elicited by active myofascial trigger points produced neck and shoulder/axillary complaints in these patients, suggesting peripheral and central sensitization in patients with postmastectomy pain. Similarly, Dibai-Filho et al. [15] reported higher intensity of myofascial pain on upper trapezius in breast cancer survivors, compared to chronic neck pain in women without breast cancer, indicating the need for a careful assessment and treatment of this pain condition. Pharmacological approach could be proposed only for a short period and in case of exacerbation of pain. Physiotherapy, specifically manual therapy, is surely a valid option to obtain better functional results and pain control, but it requires frequent sessions with a physiotherapist to be effective [10]. To design our work, we consider the study of Sharan et al. [32] that compare a group treated with physical therapies + CFA cream and a control group treated only with physical therapies and placebo cream, obtaining better results when physiotherapy was combined with CFAs cream. Various studies report the benefits of CFA topical application on knee osteoarthritis, improving ROM, pain, gait pattern, stiffness articulation, and consequentially quality of life [30–35].

In this study, a group of patients suffering from a specific cancer-related disease showed a significant pain reduction and a significant improve in mobility. The reduction in moderate disability in favor of minimal disability after 15 days (T1) is the main goal of this study, to allow women to recover in a short time and their activities of daily living and improve their quality of life. Moreover, the data of the maintenance of this result after 15 days without treatment (T2), which shows that the benefit obtained is maintained over time, is equally important.

In this specific historic period characterized by the Sars-CoV2 pandemic, access to rehabilitation and physiotherapy treatment (both in- and outpatient) has often been difficult for patients [39]; in fact, some studies in literature have suggested telerehabilitation in breast cancer survivors too [40]. Considering these difficulties, a topic treatment simple and easy to use, self-applicable at home, without side effects, has been well accepted by patients, reporting good results on their pain and neck pain-related disability.

Our study certainly has some limitations: the absence of a control group, the short follow-up, the lack of association with a physiotherapy treatment. Further studies could be needed to confirm how long the treatment is effective, to evaluate if and when the pain recurs and its intensity, and how to repeat the treatment (for a shorter or the same period of 15 days).

In conclusion, a protocol treatment based on inelastic adhesive tape with cetylated fatty acid (CFA) esters showed to be simple, effective, and easily manageable, especially when it is difficult access to rehabilitation treatment, as what happened during the pandemic in order to reduce pain and improve cervical mobility in breast cancer survivors with chronic neck pain. According to our results, this therapeutic strategy could be helpful in these patients, and also in patients with the same condition, for improvement in pain, mobility, and disability in activities of daily living.

## Data Availability

All paper documentation is available at Torre del Greco Hospital, Naples.
